# Phytochemical Identification, Acute, and Sub-Acute Oral Toxicity Studies of the Foliar Extract of *Withania frutescens*

**DOI:** 10.3390/molecules25194528

**Published:** 2020-10-02

**Authors:** Abdelfattah EL Moussaoui, Mohammed Bourhia, Fatima Zahra Jawhari, Hamza Mechchate, Meryem Slighoua, Ahmed Bari, Riaz Ullah, Hafiz Majid Mahmood, Syed Saeed Ali, Samir Ibenmoussa, Dalila Bousta, Amina Bari

**Affiliations:** 1 Laboratory of Biotechnology, Environment, Agrifood and Health, Faculty of Science, University of Sidi Mohamed Ben Abdellah, Fez BP.1796, Morocco; abdelfattah.elmoussaoui@usmba.ac.ma (A.E.M.); Jawhari.fatimazahra@gmail.com (F.Z.J.); hamza.mechchate@usmba.ac.ma (H.M.); slighoua.meryem@gmail.com (M.S.); boustadalila@gmail.com (D.B.); aminabari3@gmail.com (A.B.); 2 Laboratory of Chemistry-Biochemistry, Environment, Nutrition, and Health, Faculty of Medicine and Pharmacy, University of Casablanca, Casablanca BP.5696, Morocco; ibenmoussa@yahoo.fr; 3 Department of Pharmaceutical Chemistry and Central Laboratory, College of Pharmacy King Saud University, Riyadh 11451, Saudi Arabia; abari@ksu.edu.sa (A.B.); ssyed@ksu.edu.sa (S.S.A.); 4 Department of Pharmacognosy (MAPPRC), College of Pharmacy, King Saud University, Riyadh 11451, Saudi Arabia; rullah@ksu.edu.sa; 5 Department of Pharmacology, College of Pharmacy, King Saud University, Riyadh 11451, Saudi Arabia; harshad@ksu.edu.sa

**Keywords:** *Withania frutescens*, toxicity, biochemical parameters, histopathology, GC-MS

## Abstract

*Withania frutescens* (*W. frutescens*) is a medicinal plant widely used to treat several diseases. This work aims to study phytochemical composition as well as acute and subacute toxicity of *W. frutescens* hydroethanolic extract in mice. The phytochemical composition of *W. frutescens* extract was performed using gas chromatographic analysis. Acute toxicity was studied in vivo with oral administration of single doses 400 mg/kg, 1000 mg/kg, and 2000 mg/kg for 14 days. Subacute toxicity was studied with the administration of repeated doses of 400 mg/kg/day and 2000 mg/kg/day for 28 days. Phytochemical analysis of *W. frutescens* hydro-ethanolic extract confirmed the presence of interesting chemical compounds. Acute toxicity results showed no toxic symptoms in mice treated with an increasing dose up to a maximum of 2000 mg/kg. Alongside acute toxicity, subacute data showed no clinical symptoms nor biochemical or histological alteration in mice treated with an increasing dose up to a maximum of 2000 mg/kg compared to the control group (*p* < 0.05). This study shows no toxic effects in animals treated with *W. frutescens* extract, and, therefore, this plant can be considered safe in animals up to 2000 mg/kg under both acute and subacute toxicity conditions.

## 1. Introduction 

For a long time, plants have been used as a promising source of therapeutic agents. Currently, many developed drugs, even against cancer, could be derived from natural products or their chemically modified derivatives [1]. In recent decades, people have returned to traditional medicine including natural products due to their effectiveness in treating and preventing diseases with fewer or no side effects. For instance, the resistance of bacteria to many developed antibiotics is a dilemma that has led to using natural products from medicinal plants with more positive effects compared to synthetic drugs [2]. For several decades, medicinal plants have played a key role in pharmacological research studies and drug development. Plants contain many bioactive compounds elaborated under secondary metabolism pathways. These constituents are used as therapeutic and prophylactic agents, as raw materials for drug synthesis, or as models for pharmacologically-active compounds [3].

The genus *Withania* (Solanaceae) has been widely used as home remedies versus many ailments such as liver diseases, bronchitis, and ulcers [4,5], and as an anti-inflammatory agent through inhibition of delayed hypersensitivity [6,7]. It has also been used against anxiety, Parkinson’s disease, neurological disorders, and as a sedative agent [8,9]. Earlier data reported that this genus exhibits antioxidant properties due to its content in bioactive compounds like withaferin A, withanolide, and total polyphenols decreasing lipid peroxidation and increasing levels of suroxide dismutase [10,11]. 

Although several medicinal plants may have pharmacological activities that are beneficial to human health, they could exhibit toxic effects when ingested without scientific validity, and, therefore, the screening of potential toxicity of natural products is attention-seeking [12]. The use of natural remedies over the long-term without any evidence of health risk may indicate that a drug is harmless [13,14].

Despite the wide use of plants in traditional medicine, there is no well-developed research that can explain the toxicity of all plants used. The lack of systematic toxicity studies on *W. frutescens*, even it is largely used in alternative medicine, was the objective of the research work. 

## 2. Results and Discussion 

### 2.1. Identification of Phytochemical Compounds by GC/MS

**Figure 1:**
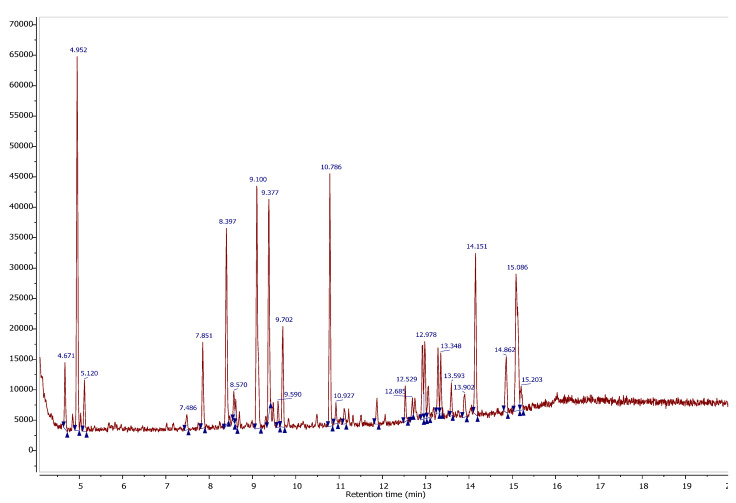
Gas chromatographic profile of *W. frutescens* extract.

Gas chromatographic profile (Figure 1) represents the peaks and the retention time of each phytochemical compound contained in the ethanolic extract studied. Each compound has a dominant percentage. The extract is mainly composed of Chromium, pentacarbonyl (13.22%), 2-phenazine carbonitrile (10.64%), Terpinenol-4 (10.04%), and 4H-1-benzopyran-4-one,2,3-dihydro-5,7-dihydroxy-2phenyl(S) (8.76%) (Table 1). The pharmacological activities of some compounds identified in the extract like ferrocene and its derivatives exhibiting anti-proliferative effects against lymphocytic leukaemia [15]. Ferrocene was considered to be a promising anti-malarial drug [16,17]. Phenazine derivatives have antimicrobial effects [18]. Besides, terpinenol-4 as a compound exists in some plants consumed by humans, which has antimicrobial effects against some microorganisms [19]. The findings of the phytochemical analysis were in agreement with the earlier found data, which showed that the extract of *W. frutescens* possessed various phytochemical compounds (tannins, mucilage, terpenoids, alkaloids, polyphenols, and tannins) [20].

### 2.2. Bodyweight

**Figure 2:**
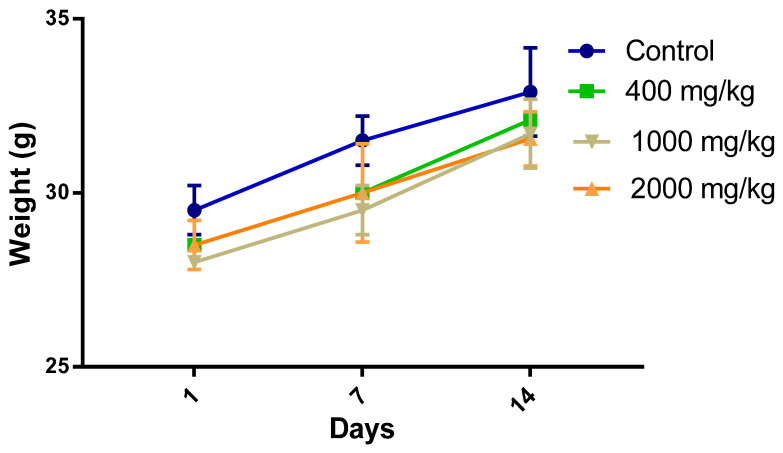
Effect of treatment with *W. frutescens* extract on body weight evolution of animals.

Bodyweight and general behavior of animals were frequently assessed to indicate the occurrence of a toxic effect or a lack of a toxic effect [21]. The oral administration of the extract to mice up to a maximum dose (2000 mg/kg) under acute toxicity conditions did not negatively affect the behavior of animals nor its body weight. All animals given the extract (400 mg/kg, 1000 mg/kg, and the dose of 2000 mg/kg) gained weight when compared to the control group (Figure 2).

The observational evaluation did not record behavioural changes for all treated groups (diarrhea, immobility, excitement, refusal of food, tremor, and mortality) during the test period. The animals treated with 2000 mg/kg acquired a relaxation that occurred in the first 40 min after treatment compared to the control group. This result was consistent with the previous literature that showed the Genus *Withania* possessed calming substances [22].

### 2.3. Subacute Toxicity

**Figure 3:**
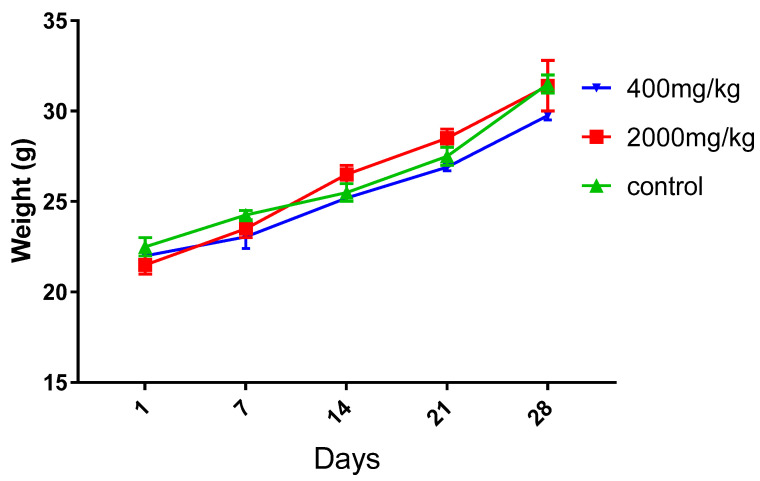
Effect of treatment with *W. frutescens* extract on body weight evolution of animals.

Physical and behavioural examination revealed no adverse effects on mice given 400 mg/kg/day and 2000 mg/kg/day when compared to the control group (Figure 3). However, all animals treated with a dose up to a maximum gained weight (*p* > 0.05). These results could indicate that the administration of leaf extract of *W. frutescens* up to 2000 mg/kg/day to mice for 28 days has no adverse effect on physical appearance and body weight. Food and water consumption by animals receiving the extract studied was not directly affected when compared to the control group (*p* > 0.05). This finding agrees with other data, which showed that humans taking the aqueous extract of *Withania somnifera* gained weight and appetite [13,23].

#### 2.3.1. Evaluation of the Organ Relative Weight

**Figure 4:**
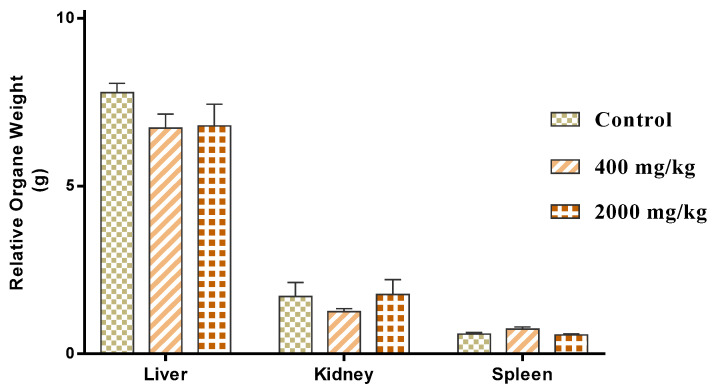
Effect of treatment with *W. frutescens* extract on relative organ weights.

After 28 days of extract administration at 400 mg/kg and 2000 mg/kg body weight (Figure 4). Vital organs such as liver, kidney, and spleen were weighed. The results showed no alteration of the organ relative weight compared to the control group (*p* > 0.05). This result could confirm the safety of *W. frutescens* extract with oral administration up to 2000 mg/kg since the modification of the organ relative weight can be induced by toxic substances [24].

#### 2.3.2. Biochemical Parameters Analysis

**Figure 5:**
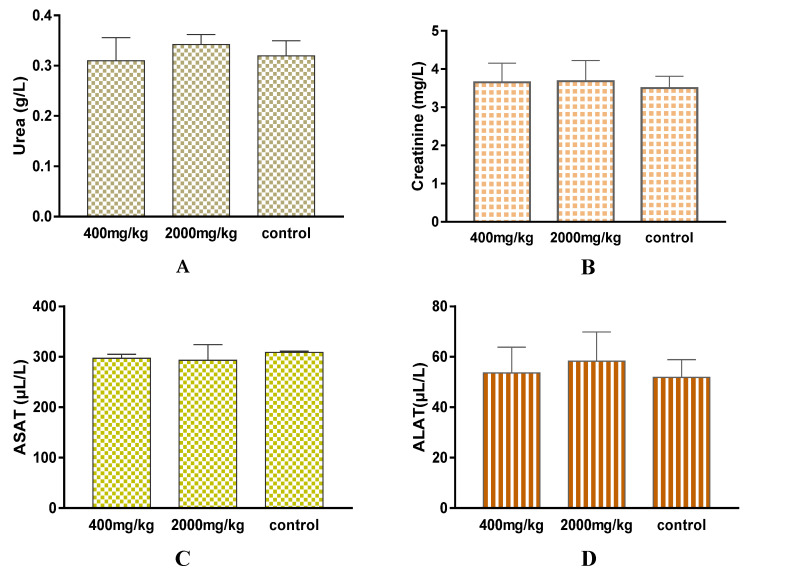
Effect of *W. frutescens* extract on animal biochemical parameters (**A**): Urea, (**B**): Creatinine, (**C**): ASAT, (**D**): ALAT. Values are expressed as mean ± SEM, *n* = 5.

The results of the biochemical parameters analyzed such as urea, creatinine, and aminotransferases (ALAT and ASAT) are shown in Figure 5. The results showed that the biochemical parameters of treated mice with a dose up to a maximum (2000 mg/kg body) were not directly affected when compared to the control mice (*p* > 0.05). These findings were in agreement with those of acute toxicity, which showed no clinical symptoms nor behavioral changes occurred in mice treated with similar doses. In the current work, potential hepatotoxicity of the extract was assessed by measuring the enzymatic activities of aminotransferases (ALAT and ASAT). An abnormal increase in aminotransferase activities (ALAT and ASAT) could frequently refer to hepatotoxicity [25]. However, there is no effect on the activity of aminotransferase of treated mice when compared to the control group (*p* > 0.05). The kidney function was also assessed for potential toxic effects induced by the extract by measuring urea and creatinine concentration since any significant change in these parameters could refer to induced-nephrotoxicity [26,27]. In addition, as a result, the findings showed no adverse effect on urea or creatinine concentration when compared to the control group (*p* > 0.05).

#### 2.3.3. Internal Organ Histology

**Figure 6:**
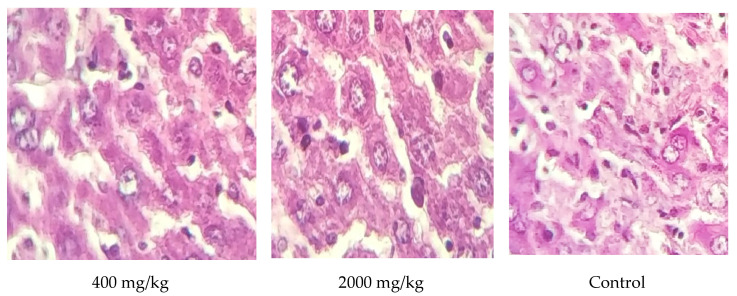
Photomicrographs of liver slices of animals treated *W. frutescens* (magnification ×40).

**Figure 7:**
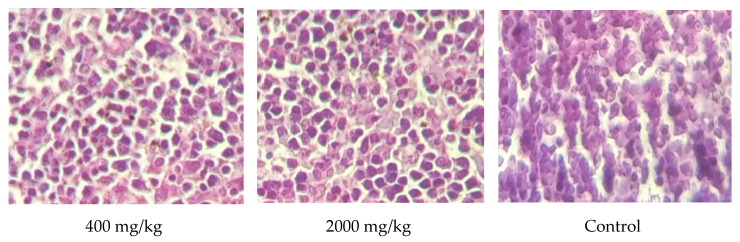
Microscopic photo of spleens of animals treated *W. frutescens* (magnification ×40).

**Figure 8:**
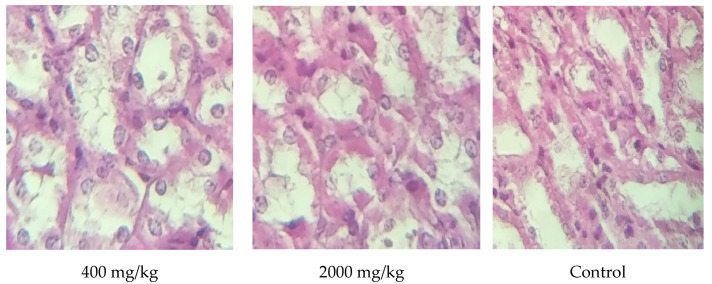
Kidney histology of animals treated with 400 mg/kg, 2000 mg/kg (magnification ×40).

Although the liver excised from all treated animals after 28 days of dosing was subjected to histological examination for analyzing the following injuries, bile ducts, hepatic vein, the artery in the portal area, and hepatic fat had no modification detected when compared to the control mice (*p* > 0.05) (Figure 6). These results were consistent with those of biochemical parameters, which showed no alteration in ASAT nor ALAT. The spleen plays a crucial role in the body to help in blood filtration in which red blood cells are recycled. The results of histological examination of the spleen tissue excised from animals treated with a dose up to a maximum of 2000 mg/kg body revealed no structural changes (Figure 7). The kidney tissues were also examined for a potential alteration of distal tubule, proximal tubule, and glomerulus. As a result, no morphological changes were noticed when compared to the control mice treated with distilled water during the whole period of dosing (Figure 8. These results were also supported by the biochemical parameters’ findings (urea and creatinine) since no modification was reported for them. These findings could confirm the safety of the extract studied up to 2000 mg/kg body since no adverse effect is noticed for the hematopoietic system [28].

The powder from leaves of *W. frutescens* has been used in folk medicine for centuries and has proven to be harmless to health. In vivo toxicity studies of products provide knowledge about safe doses in humans [29]. The results obtained in this work were used for performing a comparison with the earlier data, which showed no toxic effect occurred in animals treated with *Withania somnifera* hydroalcoholic extract under subacute toxicity conditions [28]. The standardized extract of *W. frutescens* attracts plenty of interest due to its use as an adjuvant in cancer treatment. The molecular purification of compounds contained in *W. frutescens* extract revealed that this plant contains withanoloids as an effective agent vs. cancer. Withaferin as a compound revealed in *Withania somnifera* extract showed to be a promising anticancer agent [26,29,30]. The present findings were in confirmation with the earlier literature which reported that no toxic symptoms happened in humans ingested *Withania somnifera* extract [31]. Subacute toxicity results suggest that the oral administration of *W. frutescens* extracted up to 2000 mg/kg/day does not cause any adverse effects in the animals.

## 3. Materials and Methods

### 3.1. Plant Material 

Leaves of *W. frutescens. L* were collected at the end of March 2019 from the region of Fez-Morocco. The identification of the studied plant was made by the botanist Amina Bari (Department, Sidi Mohamed Ben Abdellah University Faculty of Sciences Dhar El-Mahraz, Fez, Morocco) and the voucher specimens have been deposited in the herbarium of the faculty of sciences under the reference BPRN69. The leaves were rinsed and dried in the shade at 30 ± 2 °C. The dried leaves were crushed using an electric mixture. The resulting powder was extracted using hydro-ethanolic maceration constituted of 70% ethanol and 30% distilled water for 24 h at room temperature. Afterward, the mixture was filtered under reduced pressure using a rota-steamer. After filtration, the filtrate/extract was kept for further use.

### 3.2. Phytochemical Analysis of Plant Extract by GC/MS

The phytochemical analysis of *W. frutescens* extract was made by *N*-methyl-*N*-trimethylsilyl, silylating agent of trifluoroacetamide (MSTFA). 0.003 g of the crude extract of *W. frutescens* obtained by adding 200 μL of *N*-methyl-*N*-trimethylsilyl trifluoroacetamide (MSTFA), afterward the extract was heated at 37 °C for 30 min. 0.1 μL of this the crude extract was injected into apparatus for analysis. The analysis was effectuated using GC-MS. Model 5973 purchased from Brand Agilent Technologies. Helium was used the column carrier gas with a typical pressure range (psi) of 0.9 mL/s. Graphite furnace temperature program was made between 70–270 °C at 4 °C/min and maintained at 270 °C for further 20 min. The injector temperature was set to 280 °C and the detector temperature was set to 290 °C. The injection was made with respect to the fractionated mode.

### 3.3. Animals Used 

Mice (Swiss albinos) obtained from the Laboratory of Neuroendocrinology and Nutritional and Climatic Environment of the Faculty of Sciences Dhar El Mahraz, Fez, weighing 22 to 27 g and aged 8 weeks were used to perform the current study. Mice were typically housed in cages (five mice/cage) in environment conditions with a temperature 23 ± 2 °C and a light-dark cycle of 12 h for an acclimatization period of two weeks. The animals were fed standard pellets during the study period and had free access to water.

The procedures used to perform this study are in agreement with the international guidelines used for the use of laboratory animals. The ethical committee of the Faculty of Sciences of Fez, Morocco, revised and approved this work under the ethical clearance N-ANI-BPRN-134.

### 3.4. Preparation of Test Solutions

The crude extract obtained was dissolved in distilled water. Afterward, the mixture was stirred (3–5 min) using a magnetic stirrer. The obtained solution was kept in a refrigerator after each oral administration. The volume of solution selected for administration was determined by the following mathematical formula.

$$V=\frac{D\times P}{C}$$

*V* = volume of solution selected to be administered (mL), *D* = dose (mg/kg), *P* = weight of animal (kg), and *C* = concentration of solution selected to be administered (mg/mL).

### 3.5. Acute Toxicity 

Acute oral toxicity of the extract was evaluated in mice according to guidelines 423 [32]. After treatment, animals were observed individually at least one time during the first 30 min and regularly for the upcoming 24 h with particular attention during the first 4 h. Animals were then observed daily for 14 days. Observations focused on behavioral changes. Particular attention was paid to the observation of various manifestations of tremor, convulsions, salivation, diarrhea, lethargy, and sleeping. The body weight was also weekly measured during the whole period of dosing.

### 3.6. Subacute Toxicity 

Subacute toxicity study was designed according to the Organisation for Economic Co-operation and Development (OECD). Guidelines for the analysis of chemicals in a 28-day repeated-dose oral toxicity study in rodents [16]. For sub-acute studies, young and healthy mice were divided into three groups of five in each. Mice were given oral doses of 400 (group II) and 2000 (group III) mg/kg/day by the time the control mice were given distilled water for 28 days. Signs and symptoms of toxicity were observed during the whole period of dosing and the body weight was weekly measured for 28 days. At the end of the experimental period, all survived animals were euthanized for blood and organ collection.

#### 3.6.1. Analysis of Serum Biochemistry

Analysis of serum biochemistry was done at the end of the experiment. The collected blood from mice was transferred into tubes with anticoagulants for being centrifuged at 3500 rpm for 10 min. The plasma was recovered and stored in a freezer until further analysis. Aspartate aminotransferase (ASAT), Alanine Aminotransferase (ALAT), Creatinine, and Urea, were measured. The measurement of biochemical parameters (ASAT, ALAT, urea, and creatinine) was carried out using a Hycel Lisa 300 automaton.

#### 3.6.2. Histopathology Evaluation 

Animals were anesthetized and subjected to cerebral dislocation for vital organ collection. Kidneys, liver, and spleen were excised and weighed to calculate the relative weight. Organs excised were saved in 10% formalin for further histological analysis [17]. 

### 3.7. Statistical Analysis 

Data of the present study was expressed as means ± SEM (five replicates). Statistical significance was performed using the ANOVA test. A significant difference was considered when *p* < 0.05.

## 4. Conclusions

In light of this study, we could suggest that *W. frutescens* extract studied in terms of acute and subacute toxicity up to 2000 mg/kg/day revealed no adverse effects on mice, and, therefore, extracts from this plant are encouraged for medication up to the dose studied.

## Figures and Tables

**Table 1:** Phytochemical compounds identified in the extract by gas chromatography.

Peaks	R.T (min)	Name	Area %
1	15.203	1,7,7-Trimethylbicyclo[2.2.1]heptan-2-ol	1.19
2	15.086	Terpinenol-4	10.04
3	14.862	6,6-Dimethyl-2-methylenebicyclo[3.1.1]heptan-3-one	2.67
4	14.151	Bicyclo [3.1.1] heptan-3-ol, 6,6-dimethyl-2-methylene	6.26
5	13.902	1-Isopropyl-4-methylbicyclo[3.1.0]hexan-3-one	1.24
6	13.593	Bicyclo[3.1.0]hexan-3-one, 4-methyl-1-(1-methylethyl)	1.10
7	13.348	2,5,5-trimethylhepta-2,6-dien-4-ol	1.84
8	13.284	Cuproine	2.34
9	13.063	butanedioic acid,(trimethylsilyl oxy); bis (trimethylsilyl)	1.33
10	12.978	Malic acid, o-trimethylsilyl), bis (trimethylsilyl) ester	2.77
11	12.930	2,2′-biquinoline	3.00
12	12.685	3-oxovaleric acid	0.65
13	12.529	Hexadecanoic acid	1.17
14	11.874	Palmitic acid	1.00
15	11.115	cyclopentadieneacrylic acid	0.68
16	10.927	Ferrocene (2-carboxyethenyl)	0.75
17	10.786	4H-1-benzopyran-4-one,2,3-dihydro-5,7-dihydroxy-2phenyl(S)	8.76
18	9.702	Pyrido (3,2-d) pyrimidine-2,4(1H,3H)-dione,1,3,6,trimethyl	3.66
19	9.590	Trimethylsilyl ether of glycerol	0.86
20	9.377	2-cyanophenazine	7.67
21	9.100	2-phenazine carbonitrile	10.64
22	8.614	Cyclopentan ecarboxylic acid, 3-methyl-2-oxo, ethyl ester	0.83
23	8.570	m-phenylphenol	1.04
24	8.397	Tetramethylethylene-1,4-diol 2Tms	7.58
25	7.851	3-biphenylol	2.92
26	7.486	1-Naphtalenethiol	0.72
27	5.120	Pyradiazine,3-chloro-6-(methylthio)	1.72
28	4.952	Chromium, pentacarbonyl	13.22
29	4.671	3H-pyrazol-3-one, 2-4-dihydro-5-phenyl	2.29
